# Confirmation of the first intronic sequence in the bivalvian mitochondrial genome of *Macoma balthica* (Linnaeus, 1758)

**DOI:** 10.1098/rsbl.2022.0275

**Published:** 2022-10-05

**Authors:** Marek Lubośny, Beata Śmietanka, Rafał Lasota, Artur Burzyński

**Affiliations:** ^1^ Department of Genetics and Marine Biotechnology, Institute of Oceanology Polish Academy of Sciences, Sopot 81-712, Poland; ^2^ Faculty of Oceanography and Geography, Division of Marine Ecosystems Functioning, University of Gdańsk, Gdynia 81-378, Poland

**Keywords:** intron, mtDNA, NGS, bivalvia, DUI, rtPCR

## Abstract

In 2020, the first male-type mitochondrial genome from the clam *Macoma balthica* was published. Apart from the unusual doubly uniparental inheritance of mtDNA, scientists observed a unique (over 4k bp long) extension in the middle of the *cox2* gene. We have attempted to replicate these data by NGS DNA sequencing and explore further the expression of the long *cox2* gene. In our study, we report an even longer *cox2* gene (over 5.5 kbp) with no stop codon separating conserved *cox2* domains, as well as, based on the rtPCR, a lower relative gene expression pattern of the middle part of the gene (5′ = 1; mid = 0.46; 3′ = 0.89). Lastly, we sequenced the *cox2* gene transcript proving the excision of the intronic sequence.

## 1. Introduction

With the growing popularity of next-generation sequencing methods, scientists are discovering more new features of mitogenomes, breaking the established view of conserved structure and compactness of animal mitogenomes. Considering only animal mitogenomes, the length of mtDNA can vary greatly from the usual 16–17 k base pairs (bp) [1]. The longest known animal mitochondrial DNA belongs to clams from the Arcidae family *Anadara sativa* and reaches a length of 48 kbp (KF667521). The shortest 10 kbp long belongs to the comb jelly *Mnemiopsis leidyi* [2] (NC_016117). Mitochondrial DNA can also differ in structure from typical circular, throughout linear in some medusozoan cnidarians, calcareous sponges, ending in multipartite, fragmented in hydrozoan cnidarians, lice and jellyfish [3]. What is more, the number of protein-coding genes can also vary. There are mitogenomes lacking some of the canonical 13 protein: *atp8* in flatworms [4,5], nematodes [6,7], *atp6* and *atp8* in comb jelly [2], *atp8* and *nad5* in Hong Kong whipping frog [8], *nad5* in tuatara [9] up to mitogenomes with additional genes: *mtMuts* in Octocorallia [10]; *dnaB* in jellyfish [11]; *atp9* in sponges [12] and protein-coding novel open reading frames (*orf's*) in bivalves [13–17]. It is worth mentioning here that in some rare cases, the ‘missing’ genes might have been omitted and remain unannotated due to the low-sequence homology to their counterpart genes present in the available databases at that time (e.g. GenBank). One of those situations was reported in bivalves from genus *Mytilus,* which were thought for a long time to be missing *atp8* gene in their mtDNA [18–21]. The typical ‘average’ metazoan mitogenome does not contain any introns, but as usual in these cases, even here scientists found exceptions. Most cases concern group I and II introns found in non-bilaterian animals: hexacorals [22–26], demosponges [27–31], and placozoan [32,33]. Such taxonomic distribution is often used as empirical support for mutational-pressure hypothesis explaining lack of introns in fast-evolving mtDNA [34]. Up to this point, the only bilaterian animals where mitochondrial intronic sequences have been identified are several species of annelids [35–38].

Bivalves are one of the animal classes with a wide range of mitochondrial features scattered over many species that distinguish them from the typical structure of mtDNA. Some of them vary in mitogenome size, some possess additional tRNA*,* gene extensions, and additional protein-coding open reading frames often associated with doubly uniparental inheritance of mitochondrial DNA (DUI), which is unique for bivalves. In DUI species male individuals are heteroplasmic, possess two divergent mitogenomes (Female-type and faster evolving Male-type), while most of the female individuals are homoplasmic toward F-type mitogenome. M-type mitogenomes are localized mostly in male gonads and are typically the only mitotype passed to progeny through sperm [15,21,39–45]. Fertilized egg cells receive F-type mtDNA from mother and M-type from father, then depending on the sex of future progeny (scenario when the embryo grows into the female individual), male mitochondria get dispersed during cell divisions resulting in quick loss of detectable signal from male-type mtDNA. In the second case, when the embryo develops into a male specimen, M-type mitochondria migrate grouped together during the first cell divisions, becoming the dominant fraction in the future gonads [46–49].

The Baltic clam *Macoma balthica* (Linnaeus 1758) is an infaunal tellinid bivalve that is commonly found in marine and estuarine soft-bottom habitats in the Northern Hemisphere. The first sequencing of mitogenome of this species was performed by Saunier *et al*. [50], who assembled and annotated six nearly complete mitochondrial genomes from five mitochondrial lineages distinguished based on *cox1* haplotypes [51]. Recently, a male-type mitogenome of *Macoma balthica* was published [52]. Its unusual feature was a very long (over 4 kbp long) cytochrome c oxidase subunit II gene with stop codon separating the 5′ part of *cox2* gene. This caught our interest, so we decided to analyse the expression pattern of this gene to check if the whole sequence is expressed as a single transcript, as well as to verify if the reported stop codon that separates *cox2* domains is true or only an assembly artefact.

## 2. Methods

Individuals of *Macoma balthica*, used for DNA analysis and NGS DNA sequencing, were collected in the Gulf of Gdansk (southern Baltic Sea, Poland) using a bottom dredge on 8 November 2017. The Gulf of Gdansk is a brackish-water basin, dominated by soft-bottom habitats, with an elevated levels of pollution, eutrophication and primary production. Water temperature ranges annually from −0.43 to 24.7°C, whereas average annual water salinity is around 7–8 PSU ([53] and references therein). Collected specimens were checked for the presence of sperm and egg cells under a microscope, sectioned and stored in the freezer in 70% ethanol until DNA extraction. Individuals for RNA samples were gathered from the beach in Sopot (Poland) after the storm in February 2021 and stored frozen at −80°C. DNA extraction from male gonads was performed according to Hoarau *et al*. [54] (the protocol was described in detail in [55]). RNA was extracted from the entire body (half of the individual; approximately 1 cm × 1 cm shells) with the GenElute Mammalian Total RNA miniprep kit (Sigma). After RNA extraction, the remaining contaminating DNA was digested with DNase I (EurX) and purified again on new columns from the GenElute Mammalian Total RNA kit. To ensure the presence of chaotropic salts allowing for the binding of the nucleic acids to the columns silica resin, RNA extracts were mixed with isolation buffer in 1 : 1 ratio. Total DNA from single-male individuals was sent to Macrogen Inc. for high-throughput NGS sequencing (NovaSeq Illumina, TruSeq NGS library 2 × 150 bp). Complete mitochondrial sequences have been recovered with NOVOplasty [56] and validated by mapping NGS reads onto the assembled mitogenomes in CLC Genomic Workbench 9.5 (QIAGEN). For gene prediction and annotation MITOCONSTRICTOR [55,57] script with default parameters was used, running and combining results from CRITICA [58], Wise2 [59], GLIMMER [60], ARWEN [61], nhmmer [62] and Phobius [63] software (detailed parameters for dependent programs can be found in MITOCONSTRICTOR pythons scrips). The annotated mitochondrial genomes of *Macoma balthica* can be found in the GenBank database under MH285593 and MH285592 accession numbers. The *cox2* gene expression pattern was checked by mapping NGS RNA-seq reads (SRR5758183; SRR5758184) [64] onto the assembled mitogenome in CLC genomic workbench 9.5.

Based on the cytochrome oxidase subunit II sequence from M-type mitogenome, six pairs of primers spanning 5′-end, mid-section, and 3′-end of the sequence have been designed in Primer3 [65]. Only optimized primer pairs (with high efficiency approx. 100%) used in the study will be shown (electronic supplementary material, table S1). Primers and PCR reaction conditions were optimized on an approximately 6 kbp long PCR M-*cox2* amplicon containing complementary targets for all primers. Composition of the PCR reaction mix for the approximately 6 kb amplicon was as follows: 25 ng DNA, 0.4 U polymerase Phusion (New England Biolabs), 1 × Buffer GC for difficult templates, 0.4 µM for each primer (Lim-F1z and Lim-R3z) and 200 µM dNTP. Conditions for the PCR reaction were as follows: initial denaturation at 98°C for the 30 s then denaturation at 98°C for 10 s, annealing at 66°C for 30 s, elongation for 3 min at 72°C and final elongation 4 min at 72°C. Denaturation, annealing and elongation were repeated in 35 cycles. The products of amplification from three male individuals were then checked by gel electrophoresis and purified with the PCR/DNA Clean-up kit (EurX). Next, products were digested with the restriction enzymes (BamHI) or sent for Sanger sequencing (Genomed SA). The standard curve for rtPCR was repeated nine times in total and showed a very good correlation (five 10 × dilution points) between 10^3^ and 10^7^ target gene copies per reaction (determination coefficient *R*^2^ > 0.99) (electronic supplementary material, table S2). Parameters for all rtPCR reactions were as follows: reverse transcription at 50°C for 20 min, initial denaturation at 95°C for 10 min, followed by the 35 cycles of denaturation (at 95°C for 30 s), annealing (at 58°C for 30 s) and extension (at 72°C for 30 s). The final concentration of reagents in a single 20 µl rtPCR reaction was as follows: 1 x SG Buffer (SG OneStep RT-PCR kit; EurX), 0.4 µM of primers, 1 µl/reaction SG Enzyme Mix and approximately 20 ng of RNA template. In addition to figure 2, the rtPCR results were put in a tabularized form in electronic supplementary material, tables S3 and S4.

The nucleic acid sequence of the cytochrome oxidase c subunit II transcript was recovered in the following steps. First, the reverse transcription reactions, with optional steps for GC-rich sequences with secondary structures, were carried out according to the protocol supplied by the manufacturer (Maxima Reverse Transcriptase, Thermo Scientific). The reaction solution contained both Random Hexamer primer, Oligo(dT)_18_ primer and Lim-R3z primer. The reaction thermal profile was as follows: 10 min at 25°C, 15 min at 50°C, 10 min at 65°C, 10 min at 50°C and finally 5 min at 85°C. The first PCR reaction on cDNA template was carried out with similar conditions as for rtPCR standard curve controls (protocol above with Phusion polymerase). The only differences were the set of primers used (Lim-F1z and Lim-R3w), annealing temperature of 60°C and 30 s extension time. The PCR product was then diluted 100 × times and another nested PCR with Lim-F1z and Lim_CR22 primers was performed (Lim-F1w primer also worked but was later used in verification correct PCR product). PCR was carried out in a higher volume of 50 µl containing the following concentrations of reagents: 2 µl PCR template, 1 × TaqNova buffer, 2 mM MgCl_2_, 200 µM dNTP, 0.1 µM primers and 1 U polymerase TaqNova (Blirt). Cycling conditions were as follows: initial denaturation at 95°C, followed by 35 cycles of denaturation at 94°C for the 30 s, annealing at 52°C for 20 s, extension at 72°C for 20 s and final extension at 72°C lasted 1 min. After electrophoresis a 500–600 bp long band was cut out from TAE gel, purified with Agarose-Out DNA purification kit (EurX) and sent for Sanger sequencing (Genomed SA). Sequence analysis of mitogenomes and transcript was performed in CLC Genomic Workbench 9.5 (Qiagen) and MEGA7 [66].

## 3. Results

**Figure 1.**
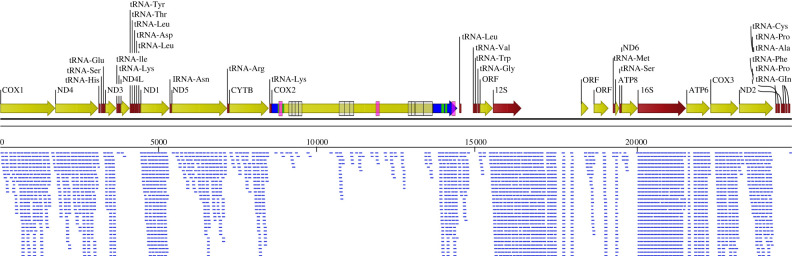
Visualization of the NGS RNA-seq reads coverage (SRR5758183; SRR5758184) over *Macoma balthica* M-type mitogenome. The blue lines in the lower part of the picture represent the coverage of NGS RNA reads, the yellow and red arrows represent mitochondrial genes, the blue arrows represent fragments of the *cox2* RNA transcript, the green boxes represent predicted transmembrane domains, the purple boxes represent sequences amplified during the rtPCR analysis and the transparent boxes represent repetitive sequences.

Based on the NGS DNA sequencing reads, we have assembled two new (F and M types) mitogenomes of *Macoma balthica* clam. During the analysis, we observed a few discrepancies between our and the published (MN528029) M-type mitogenome. The divergence is minimal (3.3% *p*-distance) and is mostly caused by the sequence and length differences in *cox2* gene and non-coding control region. The overall length of our *cox2* gene (5.8 kbp) is different, and there is no stop codon between both domains of the *cox2* gene**.** What is also interesting is that it seems almost impossible to map the RNA NGS reads (SRR5758183 and SRR5758184 [64]) onto the assembled *cox2* gene (figure 1). Only a few reads map onto the ‘inserted’ sequence fragment between 5′ and 3′ *cox2* domains. This may suggest that the assembled *cox2* with insertion is indeed an artefact created during assembly or some sort of NUMTs (nuclear mitochondrial DNA sequences) [67,68]. Another explanation why there is a discrepancy in transcript coverage with sequencing reads might be explained by a multiplicity of local secondary RNA structures or excision of the inserted fragment (intron) after transcription. To tackle this question, we took two steps back from NGS technology, designed primers located in three distinct parts of the cytochrome c oxidase II subunit and performed PCR and rtPCR reactions.

In the beginning, we checked the presence and length of *cox2*-insertion. We performed PCR spanning from the 5′ *cox2* domain to the 3′ *cox2* domain and performed digestion with the restriction enzyme BamHI (only one restriction site within the target gene). The digestion pattern correlated with the mitogenome sequence assembled in this study (two approx. 2833–2834 bp DNA fragments migrating together). In case, if the sequence reported by Capt *et al*. [52] was correct (MN528029) the expected restriction pattern would result in the 2905 bp and 1870 bp products (electronic supplementary material, figure S1). Next, we have sequenced (Sanger sequencing) the 5′-end of the PCR product looking for stop codons in the *cox2* open reading frame. No stop codon was present in the gene sequence (checked on three different individuals). The frameshift in the published sequence might have possibly been caused by the homopolymer of four out of five guanine nucleotides (electronic supplementary material, figure S2).

**Figure 2.**
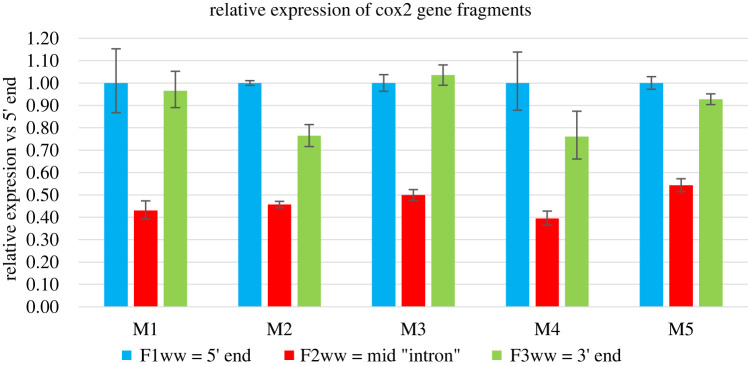
The relative expression of cox2 gene fragments. Data are presented in relation to the 5′-end of transcript ergo, relative expression for this gene to itself is 1.

With the results of the PCR, we have designed and optimized rtPCR reactions for the 5′, mid and 3′ regions trying to prove or disprove the integrity of long *cox2* transcripts. An almost equal number of transcripts for each of the three locations or a decreasing slope from 5′ through ‘mid’ to 3′ would suggest that this transcript is indeed unusually long, discrepancies in the number of mid-transcript fragments would suggest possible intron excision or (if the PCR showed shorter length) problematic assembly artefacts. Reverse transcription polymerase chain reactions on average resulted in the following relative expression pattern 5′-end = 1, mid = 0.46 and 3′-end = 0.89 (figure 2). This strong decrease in expression between the mid and both 5′ and 3′ ends supported the possibility of mid-sequence excision in an intron-like manner. Finally, to prove the presence of the intron and identify the excision sites of the transcript once and for all, we have attempted to sequence reverse-transcribed *cox2* transcript. The complete *cox2* gene and transcript sequence can be found in the electronic supplementary material, data. After alignment and incorporation of the sequenced transcript fragment into the whole gene, the acquired cytochrome c subunit II gene transcript was 1005 bp long, 234 bp from the 5′-end, and 771 bp from the 3′-end. What is interesting is that the intron splicing site is located before one of the quite conserved transmembrane domains present in F-*cox2* (*cox2* gene from female-type mitogenome) as well as in *dna*-M-*cox2* (gene from male-type mitogenome containing intronic sequence). In *rna*-M-*cox2* (*cox2* RNA transcript without the intron sequence)*,* this missing transmembrane domain is then complemented at the 3′-end of the transcript but has low-sequence homology (however keeps structural homology) to the canonical *cox2* sequence. Transcript sequencing also revealed that primers used to amplify the 5′-end of the *cox2* gene were indeed amplifying the 5′-edge of the intron.

Bioinformatic analysis of the *dna*-M-*cox2* and RNA transcript revealed the presence of a canonical intron splicing site at the 5′-end of the sequence (5′-GT) and non-canonical but still possible GG at the 3′-end of the intron (canonical AG-3′). Furthermore, the *cox2* transcript nucleotides close to the intron excision site (5′-ATCATCTTT-ACT-splicing.site and splicing.site-GG-AAAGGTGGT-3′) can create a stable 9 bp long double-stranded structure possibly facilitating connection of the 5′ and 3′ transcripts after intron excision. Unfortunately due to the length (the sequence was too long for most of the available RNA structure prediction tools) and lack of similarity to the introns described in the literature, it is difficult to reliably predict the structure of the cox2 intron, identify the reverse transcriptase coding open reading frame and assign it to any of the existing intron groups. The entire intron is in-frame so in theory any fragment of the sequence could be coding the ‘nested’ protein limited by flanking secondary RNA structures, but every BLAST search performed by us with nucleotide or translated intron sequence reported no positive hits to sequences in the GenBank database (except itself, the M-type mitogenome from *Macoma balthica*).

## 4. Discussion

Our results have some implications for the understanding of doubly uniparental inheritance in bivalves. Supernumerary open reading frames and gene extensions are often hypothesized as a key element of the molecular mechanism that stands behind DUI. On the one hand, the intronic *cox2* extension in *Macoma balthica* may not be a part of this system (there is still *m-orf* in the mitogenome), but on the other hand, we do not know if *m-cox2* may exist in more than one isoform. For example, in theory, the short one could be used most of the time in the mitochondrial energy cycle, and the long one could possibly be used during embryo development as a mitochondrial tagging mechanism. This is highly supported by a recent study on bivalve *Scrobicularia plana* done by Tassé *et al*. [69]. This clam represents a parallel situation to the one observed here with *M. balthica*, a long *cox2* gene with insertions between domains. The authors were able to detect signal from the long RNA transcript (longer than predicted), short targeted approximately 1 kb long transcript fragments containing homologous with typical *cox2* domains and inserted intron-like fragments, and big (Mw. approx. 220 kDa) protein detected with the Western blot technique. Unfortunately, the signal for a smaller 32–37 kDa version of the m-cox2 protein was not detected (personal communication). Furthermore, mitochondrial genes are expressed as polycistronic mRNA, so the presence of transcripts containing and lacking introns after excision is expected (figure 2) [69]. Of course, final proof for the existence of two m-cox2 protein isoforms in *M. balthica* cannot be reliably answered without proper proteomic studies.

There is a hypothesis explaining why the set of protein-coding genes retained in metazoan mitogenomes is limited to the genes encoding core respiratory chain subunits, stipulating that these membrane proteins are difficult to properly transport across membranes [70,71] and hence the selection favoured their *in situ* synthesis [72]. Indeed, the density of transmembrane domains within proteins encoded by the mitogenome is very high. Nevertheless, short insertions within these proteins are apparently tolerated by selection and do occur sometimes. In bivalves, such insertions are present for example in the *atp6* of giant clams [73]. The protein with a relatively small number of transmembrane domains is cox2, typically having only two such domains in a relatively long protein. Therefore, one could expect that insertions within this gene should be tolerated better. In fact there are cases in which the singular cox2 subunit was split into two separate proteins in alveolate and chlorophycean mitogenomes [74], with subsequent migration of some of the resulting genes to the nucleus. It has been also shown that fission of *cox2* occurred in an insect mitogenome of the wasp *Campsomeris plumipes* [75]. In the context of DUI bivalves, cox2 has been postulated to be involved in tagging of M type mitochondria in Unionidea [76]. In this case, the protein has a large extension with multiple additional transmembrane domains, so this interpretation seems plausible. However, there are also other, apparently independently derived, modified forms of this gene in DUI bivalves. A 100 amino acid long insertion is present in cox2 of the M-type *Meretrix lamarckii* [77], but in this case, no additional transmembrane domains were predicted in the protein. In *Arcuatula senhousia*, the gene is duplicated in M mitogenome, and one of the copies does have an additional transmembrane domain at the C-terminus [78]. In the case of *Geukensia demissa*, the M-type cox2 is singular, but also extended and features one additional transmembrane domain [79].

In conclusion, we have confirmed the first clear case of an intronic sequence in the mitochondrial genome of bivalves and one of the first in mitogenomes of bilaterian animals. This raises the question of how this discovery fits and resonates with the hypothesis that faster evolving mitogenomes do not possess introns, especially because here we have a case of even faster evolving bivalvian M-type mitogenomes.

## Data Availability

Everything is attached as electronic supplementary material [80] or available in released GenBank records mentioned in the article.
